# Applications of Diquafosol Sodium in Ophthalmology: A Comprehensive Review of Therapeutic Utility

**DOI:** 10.3390/life15030484

**Published:** 2025-03-17

**Authors:** Chelsea Qiu Lin Tan, Duoduo Wu, Xin Yun Toh, Blanche Xiaohong Lim, Kendrick Co Shih, Louis Tong, Chris Hong Long Lim

**Affiliations:** 1 Department of Ophthalmology, National University Hospital, Singapore 119228, Singapore; chelstql@gmail.com (C.Q.L.T.); wuduoduo.med@gmail.com (D.W.); blanche_lim_xh@nuhs.edu.sg (B.X.L.); 2 Lee Kong Chian School of Medicine, National Technological University, Singapore 308232, Singapore; xinyun.toh@gmail.com (X.Y.T.); louis.tong.h.t@singhealth.com.sg (L.T.); 3 Yong Loo Lin School of Medicine, National University of Singapore, Singapore 117597, Singapore; 4 Department of Ophthalmology, Li Ka Shing Faculty of Medicine, University of Hong Kong, Hong Kong, China; kcshih@hku.hk; 5 Corneal and External Eye Disease, Singapore National Eye Centre, Singapore 168751, Singapore; 6 Singapore Eye Research Institute, Singapore 168751, Singapore; 7 Center for Sustainable Medicine, Yong Loo Lin School of Medicine, National University of Singapore, Singapore 119260, Singapore; 8 Cornea and Oculoplastics Units, Department of Ophthalmology, Royal Perth Hospital, Perth 6000, Australia

**Keywords:** dry eye, diquafosol, ocular surface disorders, tear film-oriented therapy, tear film-oriented diagnosis, topical non-steroidal immunosuppressants, ocular graft-versus-host disease, soft contact lens users, LASIK, post-cataract surgery

## Abstract

Diquafosol sodium is a purinergic P2Y_2_ receptor agonist that is garnering much interest for its potential therapeutic benefits in ocular surface management. This review provides a comprehensive analysis of diquafosol’s pharmacology, clinical effectiveness, and role in the evolving landscape of ocular surface management. Future research should focus on optimising formulations, treatment duration, and exploring potential combination therapies to maximise therapeutic outcomes. By targeting underlying pathophysiological mechanisms, diquafosol represents a significant advancement in ocular surface management and a valuable addition to existing therapies.

## 1. Introduction

Dry eye disease (DED) is a chronic, multifactorial ocular surface disease [1] that is particularly common in Asia, with a pooled prevalence of 21.7% among males and 16.4% among females [2]. In China alone, a meta-analysis estimated that symptomatic DED affects approximately 31.4% of the population, corresponding to 394 million individuals [3]. DED carries a significant symptomatic burden and is associated with reduced quality of life and vision [4]. Patients with severe DED have reported that the losses in health utilities were comparable to patients on dialysis, experiencing severe angina or a disabling hip fracture [5]. At a population level, these issues translate into substantial economic costs, with estimated annual healthcare expenditures for DED reaching USD 104–167 billion in China and USD 55 billion in the United States, respectively [6,7].

Several factors contribute to the development of DED, including systemic and ocular diseases, medication intake, genetic and environmental factors and lifestyle choices. The Tear Film and Ocular Surface Society Dry Eye Workshop II (TFOS DEWS II) defines DED as a multifactorial disease of the tear and ocular surface that results in symptoms of discomfort, visual disturbance, and tear film instability, with potential damage to the ocular surface [8]. It highlights tear film hyperosmolarity and inflammation as key drivers of epithelial damage. The Asia Dry Eye Society (ADES) defines DED as a multifactorial disease characterised by an unstable tear film causing a variety of symptoms or visual impairment, potentially accompanied by ocular surface damage [9]. In contrast to TFOS DEWS II, ADES focuses on tear film instability as the primary mechanism of DED.

While their perspectives on the mechanisms leading to DED may differ, both highlight the critical role of tear film stability—the ability to maintain a smooth, continuous, and uniform layer over the corneal surface before breaking up [10]. Deficiencies in any of the key components of the tear film result in distinct DED subtypes, including aqueous-deficient, evaporative and decreased-wettability, each characterised by variations in tear film dynamics and break-up patterns [10].

Given the complex and multifactorial nature of DED, therapy should be targeted at the underlying pathogenic mechanism for maximal effectiveness. Artificial tear replacements possess a varied composition purported to optimise various DED subtypes. Novel therapeutic options of interest include topical agents such as rebamipide and perfluorohexyloctane, selenium sulphide-containing ointments, oral antioxidants, blood derivatives and office-based procedures such as vector thermal pulsation, intense pulsed light, low-level light therapy and microblepharoexfoliation [1,11,12,13]. Diquafosol sodium, a first-in-class mucin secretagogue, has introduced a paradigm shift in the treatment of DED in Asia. Although primarily indicated to improve surface wettability, its effectiveness has been demonstrated across all DED subtypes. It is one of the few therapies that enhances lipid layer thickness (LLT) and goblet cell density while reducing conjunctival epithelial damage [14]. Diquafosol has reported superior efficacy over many tear-replacement solutions in improving tear production, ocular surface staining scores and tear film stability [15,16]. Emerging research highlights diquafosol’s effectiveness in managing an array of ocular surface disorders. This review aims to summarise its utility and provide recommendations for clinical use based on existing evidence, and offer insights into future directions and innovations to optimise the management of ocular surface diseases.

## 2. Methods

**Figure 1:**
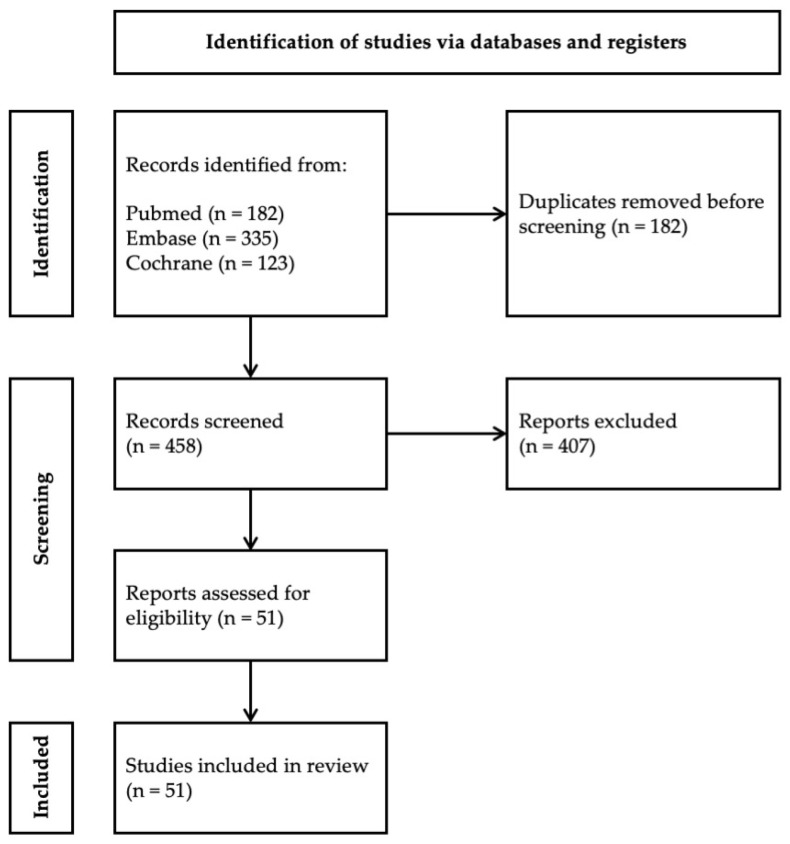
Flow chart of included articles.

The initial search from PubMed, EMBASE and COCHRANE from inception to September 2024 yielded 640 articles. After the removal of 182 duplicates, 458 articles were retrieved for abstract screening. After the exclusion of 407 articles, 51 articles were retrieved for full-text sieving and assessed for eligibility. Fifty-one studies that explored diquafosol’s utility in ocular surface management were retrieved (Figure 1). Key search terms included but were not limited to “dry eye”, “diquas”, “diquafosol”, “ocular surface disorders”, “tear film oriented therapy”, “tear film oriented diagnosis”, “tear-replacement solutions”, “topical corticosteroids”, “intense pulsed light”, “ocular graft versus host disease”, “soft contact lens users”, “LASIK” and “cataract surgery”. Both animal and human clinical studies were included in this review. Additionally, references were hand-searched for further relevant articles.

## 3. Pharmacologic Properties

### 3.1. Mechanism of Action

**Figure 2:**
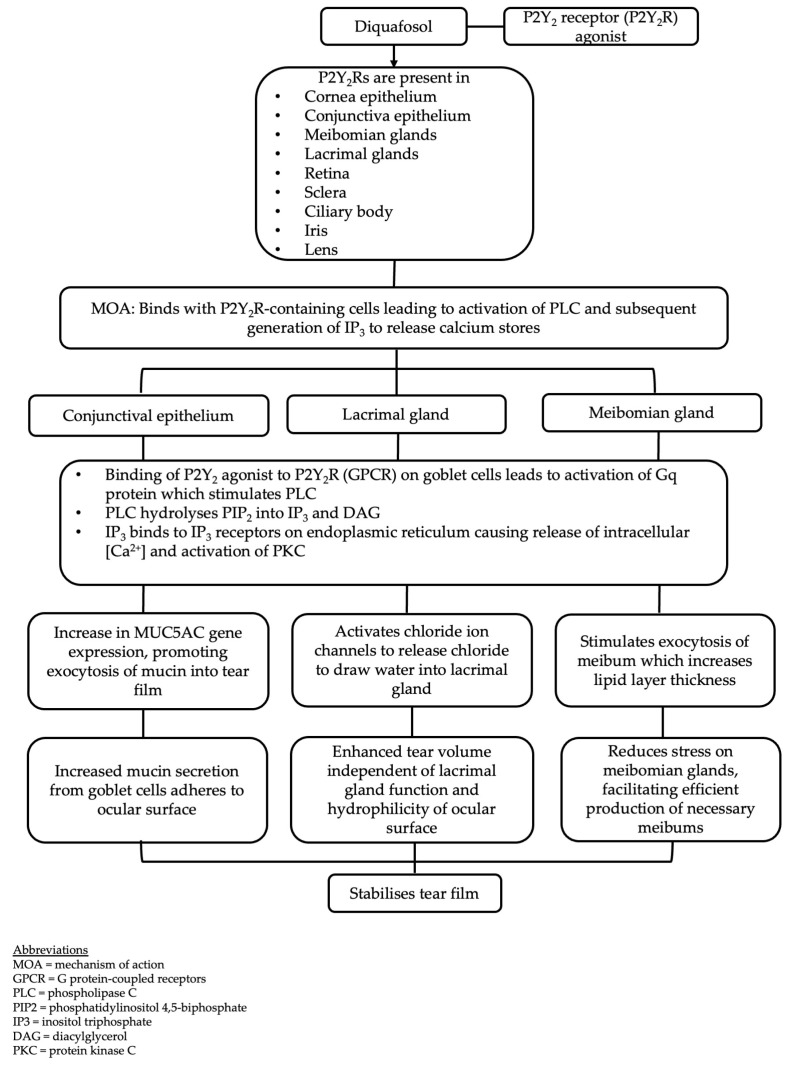
Mechanism of action of diquafosol.

Diquafosol is a stable derivative of uridine 5′-triphosphate (UTP) and a potent P2Y_2_ receptor (P2Y_2_R) agonist that plays a significant role in regulating tear and mucin secretion. In the eye and ocular adnexae, P2Y_2_Rs are expressed by the corneal and conjunctival epithelium, goblet cells and meibomian glands [17]. The activation of P2Y_2_R elevates intracellular calcium ion concentrations and facilitates fluid transport from the serosal to mucosal side via chloride channel activation to enhance tear fluid secretion from conjunctival epithelial cells and mucin secretion from conjunctival goblet cells [18]. Diquafosol also augments the gene expression of membrane-associated mucin 1 (MUC1), 4 (MUC4) and 16 (MUC16) in corneal epithelial cells and MUC1, MUC16 and membrane-associated mucin 5AC (MUC5AC) in conjunctival epithelial cells to stimulate mucin secretion [19]. Figure 2 summarises the action of diquafosol on various receptors in the ocular region to stabilise the tear film.

### 3.2. Pharmacokinetics

Information relating to the pharmacokinetic properties of diquafosol was obtained from a safety and efficacy report submitted to Japan’s Pharmaceuticals and Medical Devices Agency for regulatory approval on 5 March 2010 [20]. In a rabbit study, the ocular surface retention time of diquafosol was explored following the ocular instillation of ^14^C-diquafosol 3%, with the distribution of radioactivity across ocular tissues, including the conjunctiva and cornea, analysed. The peak radioactive concentrations were observed at 0.5 h, with a reported half-life of 17.4 h. In vitro studies with rabbit ocular tissues demonstrated that diquafosol was rapidly metabolised into uridine monophosphate, uridine diphosphate, uridine triphosphate, uridine and uracil. To evaluate systemic distribution, healthy adult volunteers received either a once-off or six-times-daily instillation of diquafosol at concentrations of 0.3%, 1%, 3% or 5% over a day. Across all the dosing regimens and concentrations tested, the diquafosol plasma levels remained below the lower limit of quantification (2 ng/mL) from five minutes to one-hour post-instillation. Plasma concentrations of its metabolites including uridine monophosphate, uridine diphosphate and uridine triphosphate remained similar to baseline, suggesting minimal systemic absorption of the drug.

### 3.3. Commercially Available Formulations

Diquafosol 3% is presently available in three commercial formulations, a formulation containing chlorhexidine gluconate solution as a preservative (Diquas^®^),a non-preservative, single-vial formulation (Diquas^®^-S) and a long-acting formulation (Diquas^®^-LX) (Santen Pharmaceutical, Japan) (Table 1). When compared against Diquas^®^, administration of Diquas^®^-S has been reported to lead to greater improvements in dry eye symptoms, tear break-up time (TBUT) and meibomian gland function across parameters such as LLT, lid margin abnormalities, meibomian gland dysfunction stage, meibum expressibility and quality and meibomian gland dropout among post-cataract surgery patients after three months of usage [21]. However, Diquas^®^-S is presently only available in a limited number of countries, with cost and logistical considerations likely limiting its commercial availability. Clinical trials of Diquafosol 2% (Prolacria™) failed to meet both their primary and secondary endpoints of achieving the clearing of fluorescein staining of the central cornea and a significant reduction in staining scores at the six-week trial endpoint compared to placebo (clinical trial: NCT00831662) [22].

### 3.4. Adverse Effects

Diquafosol is a relatively safe medication with no serious adverse events associated with its usage reported across several major clinical trials [23,24,25,26,27,28,29]. A meta-analysis estimated the odds of developing adverse events from using topical diquafosol to be 1.7 times that of hyaluronic acid (odds ratio: 1.71; 95% confidence interval (CI): 1.08 to 2.71; *p* = 0.02; I^2^ = 18%) [16]. However, reported side effects, such as ocular irritation (6.3%), discharge (2.8%), foreign body sensation (2.8%), conjunctival hyperaemia (1.4%), pain (1.4%) and pruritus (1.4%) are generally mild and transient (Table 2) [24]. An observational study by Ohashi and coworkers reported an adverse drug reaction rate of 10.7%, primarily involving non-serious symptoms of eye discharge, irritation, pain, pruritus and foreign body sensation, with the highest incidence occurring during the first month of treatment [30]. A study by Nakamura and coworkers reported that symptoms of diquafosol-induced eye irritation and pain disappeared within seven days in about 50% of cases and within 28 days in 80% of cases following continued diquafosol instillation [14]. No studies to date have evaluated the mechanisms leading to a transient worsening of DED symptoms during diquafosol initiation. It is postulated that the activation of P2Y_2_R in the acute phase induces a local pro-inflammatory state over the ocular surface [31]. Additionally, on compromised ocular surfaces with corneal epithelial erosions, diquafosol may bind to P2Y_2_R in nerve terminals and P2X3 purinergic pain receptors to induce pain and discomfort [31]. Therefore, the authors recommend the active management of ocular surface inflammation prior to the commencement of topical diquafosol for DED. Additionally, patients ought to be counselled about possible ocular discomfort and the worsening of dry eye symptoms upon the commencement of diquafosol and encouraged to persist with treatment if minor side effects manifest.

Systemically, research has reported P2Y_2_R to worsen inflammation [31]. The activation of P2Y_2_R is reported to induce pro-inflammatory states in gastrointestinal, respiratory and neurological cells, and has also been implicated in cancer pathogenesis [32,33,34]. However, P2Y_2_R agonists have also been demonstrated to promote wound healing through the recruitment of leukocytes to sites of tissue damage, facilitating the differentiation and proliferation of structural cells [35]. Nevertheless, topical administration of diquafosol is unlikely to yield any systemic adverse reactions as it is rapidly metabolised at the ocular surface. The random sampling of 25 patients receiving topical diquafosol for six months showed no elevations in the systemic concentration of derived metabolites relating to diquafosol [36]. Thus far, no studies have evaluated its safety during pregnancy.

### 3.5. Effects on Tear Stimulation

Diquafosol administration has been reported to increase tear fluid secretion in murine dry eye models. A study conducted on Goto-Kakizaki rats, a spontaneous murine model of type 2 diabetes with corneal neuropathy, which were exposed to constant airflow towards the face reported that diquafosol administration significantly increased Schirmer’s test results after 15 min (*p* < 0.01) and reduced corneal fluorescein staining scores at four (*p* < 0.05) and six weeks (*p* < 0.01) [37]. Exact values were not reported by the authors. This suggests the possible role of diquafosol in stimulating tear production, despite the presence of impaired corneal neuronal function. Another in vivo murine study investigated the effect of diquafosol administration on corneal epithelial defects, with the percentage of wound closure monitored via image analysis [38]. Rats were anesthetised followed by the creation of a 3 mm central corneal epithelial defect with a burr. The instillation of diquafosol facilitated earlier epithelial healing compared to control eyes at 12 and 24 h, respectively (percentage wound closure of diquafosol-treated eyes at 12 and 24 h: 63.4 ± 2.0% and 98.1 ± 1.1%; percentage wound closure of control eyes at 12 and 24 h: 42.7 ± 2.5% and 82.3 ± 3.2%). However, no mention of the use of topical agents or vehicles in the control group was reported. This is further supported by in vitro experiments demonstrating that diquafosol induces extracellular signal-regulated kinase cell proliferation, epithelial growth factor receptor phosphorylation and increased intracellular calcium, suggesting that the accelerated corneal epithelial healing process may be attributed to intracellular calcium-mediated epithelial growth factor receptor signalling pathways through the activation of P2Y_2_R [38].

Studies in human subjects have reported similar improvements in tear secretion. The administration of diquafosol in both healthy and DED patients demonstrated an increased radius of curvature of the central lower tear meniscus for up to 30 min after instillation [39]. The radius of curvature of the lower tear meniscus shares a linear relationship with the cumulative tear volume over the ocular surface, serving as a surrogate measure of aqueous tear volume [40,41]. This was also observed in patients with aqueous-deficient DED secondary to Sjögren’s syndrome, where the instillation of diquafosol significantly increased the central lower tear meniscus radius curvature 15 min post-instillation [42]. These results suggest the role of diquafosol in promoting tear fluid secretion from conjunctival epithelial cells, independent of lacrimal gland function [38,42].

### 3.6. Effects on Lipid Secretion

Diquafosol has also been demonstrated to stimulate holocrine-like lipid secretion from meibocytes via the activation of P2Y_2_R. Tissue sections of meibomian gland specimens from superoxide dismutase-1 (Sod1) mice and wild-type (WT) mice were stained with Oil Red O stain and analysed digitally using image processing software (Axioplan 2 Imaging, Carl Zeiss, Jena, Germany and Adobe Photoshop, San Jose, CA, USA) to quantify pixels corresponding to lipid droplets [43]. The administration of diquafosol six times daily for two weeks increased the total number of lipid droplets (from 20 ± 15 droplets pre-treatment to 100 ± 80 droplets two weeks post-treatment, *p* < 0.01, in Sod1 mice and from 25 ± 10 droplets pre-treatment to 60 ± 100 droplets two weeks post-treatment, *p* < 0.05, in WT mice), which was attributed to increased meibum secretion. There were also documented improvements in corneal fluorescein staining score, attributed by the authors to improved tear film stability and the impact of diquafosol on corneal epithelial healing. This was supported by an in vitro study of cultivated rabbit meibomian gland cells, suggesting that diquafosol induced intracellular calcium signalling in a dose-dependent manner, increasing total cholesterol cellular release [44]. Clinically, the administration of diquafosol in human eyes increased LLT and tear film stability. Interferometry measurements of tear film LLT in normal human eyes showed a statistically significant increase in mean thickness from 62.3 ± 31.1 nm to 77.0 ± 39.5 nm (*p* < 0.001), 79.3 ± 40.5 nm (*p* < 0.001) and 77.7 ± 43.6 nm (*p* = 0.009) at the 15, 30 and 60 min marks, respectively [45]. A study among dry eye patients revealed similar improvements in LLT, from an initial 49.4 ± 16.2 nm to 70.6 ± 28.2 nm (*p* < 0.001) and 63.9 ± 30.0 nm (*p* = 0.042) at 30 and 60 minutes after diquafosol instillation [46]. In comparison, the instillation of artificial tears did not result in a significant increase in LLT after treatment, with measurements reported at 52.9 ± 22.8 nm, 52.3 ± 20.3 nm and 50.3 ± 19.8 nm at 30, 60 and 90 min, respectively (*p* > 0.05 for all) [46].

### 3.7. Effects on Mucin Secretion

Diquafosol is primarily a mucin secretagogue that improves corneal wettability by stimulating the expression of human epithelial mucins via intracellular extracellular signal-regulated kinase [19]. Mucins are categorised into two groups: membrane-associated mucins and goblet-cell-secreted mucins. MUC1, MUC4 and MUC16 are produced by both the corneal and conjunctiva epithelia, while MUC5AC is a secretory mucin produced by goblet cells [47]. Membrane-associated mucins form a dense protective glycocalyx barrier over the ocular surface epithelia, facilitating debris clearance and improving the lubricity effect of the tear film, thereby reducing the friction generated between the eyelid and cornea interfaces [48,49]. Secretory mucins assist in clearing ocular surface pathogens and improve surface lubricity by forming a highly hydrated mucus gel that reduces friction [49,50]. In a rabbit study comparing the effects of diquafosol, rebamipide and artificial tears, only diquafosol was found to increase MUC5AC levels in rabbits’ tears after 15 min (*p* < 0.01) [51]. Similar observations in canine models have been documented, with statistically significant increases in tear film MUC5AC concentrations at 300 min (*p* = 0.033) following the administration of diquafosol, although neither Schirmer’s test nor phenol red thread testing demonstrated any statistically significant differences [52]. Another study by Lee and coworkers demonstrated an increased expression of MUC1 and MUC16 by human conjunctival epithelial cells following the instillation of diquafosol [19]. These in vivo and in vitro studies support the efficacy of diquafosol in inducing the secretion of membrane-associated and secretory mucins on the ocular surface.

## 4. Therapeutic Efficacy

Table 3 summarises the results and conclusions of the studies included in this review for various DED subtypes.

### 4.1. Dry Eye Disease

Multiple studies have reported the efficacy of diquafosol in alleviating the symptoms and signs of DED. Ohashi and coworkers reported significant symptomatic improvements and reductions in Dry Eye-Related Quality of Life Score (DEQS) values in patients receiving topical diquafosol for a duration of 12 months [30]. Randomised double-masked parallel-group trials also reported findings of greater improvements in foreign body sensation and eyelid heaviness among patients receiving diquafosol compared to placebo artificial tears [24,25]. A randomised controlled trial involving patients with DED reported comparable improvements in corneal fluorescein staining score (−2.1 ± 1.5 in diquafosol, −2.0 ± 1.3 in sodium hyaluronate at week four; 95% confidence interval (CI): −0.303 to 0.181) between patients who utilised diquafosol compared to sodium hyaluronate eyedrops (*p* > 0.05) [26]. However, improvements in conjunctival rose bengal staining score were more significant among diquafosol users (−2.5 ± 2.0 in diquafosol, −2.0 ± 1.9 in sodium hyaluronate at week four; *p* = 0.019). Both treatment groups experienced improvements in TBUT, by 1.046 ± 1.797 s and 0.832 ± 1.775 s, respectively, with no statistically significant differences between findings [26]. Meta-analyses of randomised controlled trials exploring the outcomes of diquafosol administration for the treatment of DED have reported its efficacy in alleviating subjective ocular symptoms and improving dry eye indices compared to artificial tears [16,53]. Sun and co-workers analysed nine randomised controlled trials that recruited patients with DED and reported significant improvements in Ocular Surface Disease Index (OSDI) (mean difference (MD): −3.59; 95% CI: −4.68 to −2.50; *p* < 0.001; I^2^ = 6%), Schirmer’s test (MD: 1.08 mm; 95% CI: 0.41 to 1.76; *p* = 0.002; I^2^ = 0%), TBUT (MD: 0.60 s; 95% CI: 0.20 to 0.99; *p* = 0.003; I^2^ = 63%), corneal fluorescein staining score (MD: −0.20; 95% CI: −0.37 to −0.03; *p* = 0.02; I^2^ = 58%) and rose bengal staining score (MD: −0.62; 95% CI: −0.88 to −0.35; *p* < 0.001; I^2^ = 15%) [16]. A cross-sectional study by Nam and coworkers in patients with normal eyes demonstrated that the administration of diquafosol resulted in improvements in measured TBUT from 4.03 ± 1.04 s at baseline to 5.53 ± 1.43 s (*p* = 0.005), 5.31 ± 1.26 s (*p* = 0.005), 4.65 ± 1.12 s (*p* = 0.022) and 4.08 ± 1.07 s (*p* = 0.959) at 5, 10, 15 and 20 min, respectively, compared to hyaluronic acid, with measurements of 4.15 ± 0.98 s at baseline to 4.16 ± 1.36 s (*p* = 0.953), 3.78 ± 1.44 s (*p* = 0.285), 3.12 ± 1.15 s (*p* = 0.022) and 2.95 ± 0.91 s (*p* = 0.007) at 5, 10, 15 and 20 min, respectively [54].

Diquafosol has also been shown to inhibit nuclear factor kappa B (NF-kB) signalling and other inflammatory factors induced by hyperosmotic stress in in vitro studies using human corneal epithelial cells, suggesting its role in the management of inflammation in DED [81]. A study conducted on DED patients receiving cyclosporine 0.1%, cyclosporine 0.05% or diquafosol measured the downregulation of tear proteomes (AFM, ALCAM, CFB, H1-4, PON1, RAP1B and RBP4) across all three groups after 12 weeks of treatment, suggesting a reduction in inflammation [55]. Clinical parameters, including conjunctival fluorescein staining score and TBUT, improved following the administration of all the medications compared to baseline. However, corneal fluorescein staining scores improved more significantly in patients receiving cyclosporine compared to diquafosol from baseline to 12 weeks (cyclosporine 0.1%: 4.89 ± 1.26 to 1.39 ± 1.37, *p* < 0.001; cyclosporine 0.05%: 4.69 ± 1.12 to 1.31 ± 1.52, *p* < 0.001; diquafosol: 4.31 ± 0.93 to 2.15 ± 1.42, *p* < 0.001). A prospective non-randomised observational study of patients with DED comparing the administration of only cyclosporine 0.1% once daily versus cyclosporine 0.1% once daily in combination with diquafosol six times daily reported that the combination therapy led to a greater degree of improvement in TBUT compared to patients receiving only cyclosporine 0.1% (combination: 2.13 ± 2.41 s vs. 1.07 ± 1.71 s; *p* = 0.001) [56]. Therefore, the administration of diquafosol and cyclosporine in combination appears to produce synergistic effects over the ocular surface. Thus far, evidence remains inconclusive on whether patients with a poor response to artificial tears should be preferentially started on topical diquafosol or cyclosporine. A study exploring patients’ considerations and preferences around medications in DED management has reported that costs and side effect profiles are major attributing factors to the choice of therapy [82]. While diquafosol offers a more favourable side effect profile, physicians should be mindful that the initiation of diquafosol in the early phases, particularly in patients with significant inflammation, may exacerbate ocular surface symptoms. This benefit must be weighed carefully against diquafosol’s cost and the need for more frequent administration compared to cyclosporine.

### 4.2. Meibomian Gland Dysfunction

Meibomian gland dysfunction is a chronic process characterised by structural abnormalities of the meibomian gland, terminal duct obstruction and changes in meibum quality [83]. A reduction in meibum secretion and changes to both its viscosity and lipid composition disrupts the tear film lipid layer, contributing to increased evaporative loss. Several studies have identified the potential of utilising diquafosol in the management of patients with meibomian gland dysfunction. A longitudinal study of patients with obstructive meibomian gland dysfunction receiving diquafosol four times daily for four months or more reported an improvement in measured mean meibomian gland area from 36.9 ± 10.1% pre-treatment to 41.5 ± 9.2% post-treatment (*p* < 0.001) [57]. Ocular surface symptoms, lid margin abnormalities, such as the plugging of meibomian orifices, and TBUT also improved after the instillation of diquafosol. Moreover, diquafosol has been reported to significantly increase LLT [45]. A study analysing DED patients receiving either topical diquafosol, normal saline, sodium hyaluronate 0.1% or gatifloxacin 0.3% found that only diquafosol led to a significant increase in LLT (mean change in LLT from baseline to 20 min after instillation of eyedrops: 12.6 ± 2.0 nm for diquafosol (*p* < 0.001), 1.2 ± 2.2 nm for normal saline (*p* = 0.301), 1.5 ± 2.0 nm for sodium hyaluronate (*p* = 0.495) and 0.5 ± 3.2 nm for gatifloxacin (*p* = 0.884)) [58]. These results suggest a possible therapeutic role for diquafosol in the treatment of evaporative DED.

### 4.3. Aqueous-Deficient Dry Eye Disease

Aqueous-deficient DED is a subtype of DED characterised by decreased tear production by the lacrimal and accessory glands, affecting up to one-third of patients diagnosed with DED [84]. Aqueous-deficient DED can arise due to a plethora of pathological mechanisms that can ultimately result in lacrimal gland damage. Donthineni and coworkers have suggested a classification comprising four major groups: immune-mediated lacrimal gland inflammation (such as Sjogren’s syndrome), conjunctival cicatrisation (mucous membrane pemphigoid and Stevens–Johnson syndrome), neurogenic causes (for example diabetes mellitus) and lacrimal gland loss (arising from trauma, age or congenital alacrima) [85].

Diquafosol has been shown to be effective in alleviating both subjective and objective clinical parameters of aqueous-deficient DED. A study recruiting patients with mild-to-moderate aqueous-deficient DED reported that patients receiving diquafosol experienced significant improvements in subjective dry eye symptoms evaluated by the authors using a composite severity scoring system (exact scores were not reported, *p* < 0.01), corneal fluorescein staining score (4.1 ± 1.8 to 1.1 ± 1.1, *p* < 0.01) and tear meniscus height (126 ± 24 μm to 171 ± 48 μm, *p* < 0.01), with no major adverse reactions reported [59]. The use of diquafosol has also been reported to improve the optical quality of the tear film, with higher-order aberrations used as a surrogate measurement in patients receiving diquafosol for four weeks. These patients experienced improvements in higher-order aberrations measured from a wavefront sensor at four weeks compared to baseline (0.180 ± 0.06 μm to 0.148 ± 0.039 μm, *p* = 0.035), with improvements in subjective dry eye symptom scores (24.3 ± 6.7 to 15.3 ± 6.0, *p* < 0.001), corneal fluorescein staining scores (4.6 ± 1.4 to 1.9 ± 1.1, *p* < 0.001) and TBUT (1.6 ± 0.8 s to 3.1 ± 0.8 s, *p* < 0.001), although no significant improvements were demonstrated with conjunctival fluorescein staining score (5.3 ± 1.3 to 4.8 ± 1.1, *p* = 0.078) and Schirmer’s test (1.7 ± 2.0 mm to 1.2 ± 1.8 mm, *p* = 0.228) [60].

Patients with Sjogren’s syndrome also benefit from diquafosol, with its use associated with a significant increase in central lower tear meniscus radius curvature 15 min after diquafosol instillation (0.16 ± 0.07 mm to 0.21 ± 0.08 mm, *p* < 0.001), while no improvements were reported in patients receiving solely artificial tears [42]. Another observational study of female patients with Sjogren’s syndrome who were symptomatic despite treatment with artificial tears reported that the addition of diquafosol to the existing treatment regimen resulted in improvements in both subjective symptoms as well as objective parameters, including tear meniscus radius (baseline vs. 12 months: *p* < 0.001), TBUT (baseline vs. 12 months: *p* < 0.05), corneal fluorescein staining score (baseline vs. 12 months: *p* < 0.001) and conjunctival fluorescein staining score (baseline vs. 12 months: *p* < 0.05), at regular intervals up to the 12th month of diquafosol treatment [61]. Importantly, all patients in the study who experienced symptomatic flares were also permitted to instil topical corticosteroids up to twice daily, which may have confounded these findings. However, the proportion of patients receiving topical corticosteroids was not reported.

### 4.4. Ocular Graft-Versus-Host Disease (oGVHD)

Graft-versus-host disease (GVHD) is a complex immunological condition that arises from donor T-cell-mediated responses towards recipient antigens, inciting tissue damage and the cytokine-mediated activation of antigen-presenting cells [86]. Chronic GVHD involves additional processes such as thymic injury, B-cell autoantibody synthesis, and the formation of profibrotic lesions [87]. oGVHD represents a significant complication of GVHD, with cicatrisation and desiccation increasing the risk of corneal ulceration and perforation. oGVHD may affect all tear film components. Alloreactive T-cells promote myofibroblast proliferation which leads to fibrosis and the destruction of lacrimal glands, with a consequent reduction in aqueous tear production [88]. T-cell infiltration, endothelial injury, neovascularisation and fibroblast activation have been observed in the meibomian glands of murine chronic GVHD models [89]. Another study reported that allogenic GVHD murine models had significantly higher meibomian gland plugging scores (1.5 vs. 0, *p* < 0.001) and more noticeable meibomian gland atrophy, scored on the meiboscale (93% vs. 47% atrophy), compared to the control group with no purified splenic T-cells. These results suggest that meibomian gland changes occur with oGVHD which can reduce both the quantity and quality of meibum secretion in stabilising the lipid tear film layer [90]. Furthermore, mucin secretion is also impaired in oGVHD patients. Ogawa and coworkers suggest that epithelial–mesenchymal transition, characterised by the replacement of epithelial cells with mesenchymal markers, incites the basal epithelial secretion of abnormal collagen bundles, contributing to conjunctival fibrosis that damages the mucin secretory function of goblet cells [91]. This is supported by findings in murine GVHD models, in which a reduction in both the area and thickness of the corneal glycocalyx has been reported [92]. Cicatrising changes that lead to eyelid abnormalities such as ectropion, entropion, lagophthalmos and trichiasis can further compromise the ocular surface [88].

Diquafosol has been studied as a treatment for oGVHD primarily for its mucin-secreting properties, but has also been postulated to augment other components of the tear film. In a retrospective study of patients with mild-to-moderate chronic GVHD-induced DED using diquafosol for up to 17 months, marked improvements in corneal fluorescein staining score (5.9 ± 0.6 to 1.3 ± 1.1, *p* < 0.001), corneal and conjunctival rose bengal staining score, an aggregate score of staining for the cornea, temporal and nasal conjunctiva (4.7 ± 1.6 to 2.0 ± 1.5, *p* = 0.008), and TBUT (2.6 ± 0.9 s to 4.6 ± 1.6 s, *p* = 0.009) were reported [62]. An anecdotal report of a 61-year-old woman with severe DED secondary to chronic GVHD showed that a combination therapy of topical diquafosol six times daily with topical rebamipide four times daily resulted in a substantial reduction in an aggregated visual analogue score which measured 12 symptoms—asthenopia, pain, discharge, foreign body sensation, epiphora, burning, ocular itching, dull sensation, conjunctival injection, dullness, dryness and photophobia—from 33 points at baseline to 8.5 points. Improvements in objective clinical findings include corneal fluorescein staining score (4 to 1.5), cornea and conjunctiva rose bengal scores, an aggregate of nasal, temporal conjunctiva and cornea staining (4 to 1) and TBUT (2.3 s to 10 s) [63]. However, no improvement in Schirmer’s test (3.0 mm to 3.0 mm) was identified compared to baseline. Further large-scale studies are required to ascertain diquafosol’s effectiveness in treating oGVHD.

### 4.5. Glaucoma Medication- and Preservative-Related Ocular Surface Disease

It has been reported that up to 59% of glaucoma patients experience ocular surface symptoms, with 78% of patients in an examined cohort exhibiting signs of ocular surface disease [93]. The pathogenesis of this is multifactorial, but has been significantly attributed to medicamentosa arising from the active ingredients, its excipients and associated preservatives [94]. Beta blocker formulations, particularly timolol maleate, have been reported to disrupt tear film stability, reduce tear secretions and incite cicatrising changes over the conjunctival epithelium [95,96,97,98,99]. Prostaglandin analogues induce obstructive meibomian gland dysfunction, which exacerbates ocular surface disease and may contribute to poor compliance with glaucoma therapy [97,100]. Moreover, prostaglandin-associated periorbitopathy can induce periorbital and eyelid changes, such as trichiasis and periorbital fat loss, that further alter and compromise the ocular surface [101]. Benzalkonium chloride (BAK) is a commonly used preservative that has been demonstrated to disrupt tear film homeostasis and alter corneal sensitivity in a dose-dependent manner [102,103]. A study examining corneal sensitivity in glaucoma patients using BAK-containing eyedrops reported a reduction in corneal sensitivity, measured with a Cochet–Bonnet esthesiometer, in a dose-dependent fashion. The findings for corneal sensitivity were 56.2 ± 5.2 mm, 50.3 ± 12.5 mm and 44.3 ± 13.6 mm among those treated with none, one and two or more instillations of preserved eyedrops, respectively [104]. In vivo confocal microscopy has also been applied, which demonstrates a significant reduction in sub-basal corneal nerve density among patients treated with BAK-containing medications as compared to BAK-free formulations [105]. BAK has also been shown to incite corneal, conjunctival, meibomian gland and trabecular meshwork cell toxicity [106,107,108]. A reduction in goblet cell density following a brief exposure of the ocular surface to BAK-containing formulations has been demonstrated using impression cytology [109]. In vitro studies with human corneal–limbal epithelial cell cultures showed that prolonged exposure to 0.0025% and 0.01% BAK for an hour decreased the amount of functional mucin, eventually causing the complete destruction of the mucous layer and diffuse damage to superficial corneal epithelial cells [110]. The instillation of BAK-preserved latanoprost upregulates tear inflammatory cytokine levels, including IL-2, IL-5, IL-10, IL-12 (p70), IL-13, IL-15, IL-17, basic fibroblast growth factor and platelet-derived growth factor, compared to preservative-free formulations of latanoprost [111]. Several clinical studies have reported an association between BAK-containing medications and both worsening OSDI scores and poorer glaucoma filtering surgery outcomes [112,113]. It has been postulated that BAK induces conjunctival subepithelial inflammation and fibrosis [114] which aggravates the wound healing process in glaucoma filtering surgeries [115].

Diquafosol has been suggested to be useful in the management of glaucoma medication- and preservative-related ocular surface symptoms due to its ability to promote tear and mucin secretion and stimulate meibomian gland function. Diquafosol contains chlorhexidine at a concentration of 0.0001–0.1% as a preservative, as detailed in the product insert. Chlorhexidine gluconate has been found to be safe and effective as an ocular surface antiseptic at concentrations between 0.05 and 0.1% [116]. While higher concentrations of chlorhexidine gluconate at 2–4% have been shown in rabbit studies to exhibit dose-dependent toxic effects such as corneal epithelial oedema, corneal de-epithelisation, conjunctival chemosis, bulbar conjunctival hyperaemia and anterior stromal oedema [117,118], the application of concentrations of 1% or less did not result in delays in corneal re-epithelisation among experimental rabbit corneal abrasion models, with mild conjunctivitis being the only complication reported [119]. This suggests that diquafosol, even in its chlorhexidine gluconate preservative-containing formulation, is safe and produces minimal preservative-induced ocular surface complications. A clinical study recruited glaucoma patients suffering from DED and reported improvements in mean OSDI score (52.17 ± 13.02 to 48.77 ± 13.27, *p* = 0.041), TBUT (3.79 ± 1.94 s to 4.70 ± 2.81 s, *p* = 0.009) and Schirmer’s test results (4.52 ± 2.11 mm to 5.64 ± 2.79 mm, *p* = 0.001) up to 52 weeks following the initiation of diquafosol [64]. Impression cytology demonstrated a sustained increase in goblet cell density which began four weeks after commencing diquafosol and lasted up to the 52-week mark (baseline: 445.1 ± 92.2 cells/mm^2^; 4 weeks: 511.0 ± 110.8 cells/mm^2^; 12 weeks: 520.5 ± 121.8 cells/mm^2^; 36 weeks: 504.8 ± 160.3 cells/mm^2^; 52 weeks: 512.4 ± 177.3 cells/mm^2^; *p* < 0.05 for all). There was, however, no information provided regarding the type of anti-glaucoma medication used. Another study consisting of normal-tension glaucoma patients who were either on a preservative-containing prostaglandin analogue, preservative-free prostaglandin analogue or a combination of BAK-containing prostaglandin analogue (BAK-PGA) and diquafosol (BAK-PGA  +  DQS) suggested that diquafosol was protective against the meibomian gland dropout associated with BAK-PGA [65]. In the BAK-PGA group, meibomian gland dropout increased significantly at 9 and 12 months after initiation of topical therapy compared to baseline, while no statistically significant changes were observed among patients using solely BAK-free formulations or a BAK-PGA + DQS combination. Additionally, another study by Liu and coworkers on patients who underwent glaucoma surgery demonstrated that diquafosol administration in the early postoperative period following trabeculectomy improved measured tear meniscus height (0.24 ± 0.16 mm to 0.28 ± 0.15 mm, *p* = 0.025), TBUT (5.08 ± 3.01 s to 9.02 ± 3.20 s, *p* < 0.05) and LLT (4.13 ± 1.36 nm to 4.81 ± 1.45 nm, *p* = 0.007) at eight weeks postoperation compared to baseline [66].

### 4.6. Cataract Surgery

Cataract surgery has been associated with a worsening of tear film parameters and increased dry eye symptoms postoperatively. A study examined patients three months after undergoing cataract surgery and demonstrated statistically significant deteriorations of tear film parameters and increased dry eye symptoms [120]. Miyake and coworkers examined patients at one month after cataract surgery and reported that 31% of patients met the criteria for a diagnosis of DED [121]. This has been attributed to a range of factors, including medicamentosa, the surgical environment; such as phototoxic effects from the operating microscope and surface desiccation, and manipulation [122,123,124,125,126,127]. In patients with pre-existing ocular surface compromise, cataract surgery can alter meibum quality, exacerbate tear film instability and worsen symptoms [128].

In a study comparing patients receiving preservative-free diquafosol, preservative-containing diquafosol and preservative-free sodium hyaluronate commencing postoperatively from day one for three months, both groups of patients receiving diquafosol experienced significant improvements in measured TBUT at one month postoperation compared to their preoperative values (preservative-free: 4.6 ± 2.2 s to 6.3 ± 3.6 s, *p* < 0.001; preservative-containing: 5.0 ± 2.5 s to 7.0 ± 2.8 s, *p* < 0.001) [21]. These findings were compared to patients receiving sodium hyaluronate, where a decrease in TBUT from 4.6 ± 1.8 s to 3.7 ± 1.4 s (*p* = 0.038) at one-month post-surgery was identified. At three months postoperation, only patients receiving preservative-free diquafosol maintained significant improvements in TBUT compared to preoperative values (6.5 ± 3.5 s vs. 4.6 ± 2.2 s, *p* = 0.038), with a significantly increased TBUT compared to patients receiving sodium hyaluronate (4.7 ± 2.3 s vs. 4.6 ± 1.8 s, *p* = 0.038). Similarly, OSDI and corneal fluorescein staining scores significantly improved in all patients receiving diquafosol compared to sodium hyaluronate. Schirmer’s test results remained unchanged across all the groups throughout the study period. A study demonstrated that diquafosol administered six times daily postoperatively after cataract surgery resulted in significantly improved TBUT (4.88 ± 2.52 s to 6.69 ± 2.23 s at three months, *p* < 0.001), corneal fluorescein staining scores (values at three months were not reported, *p* = 0.045) and conjunctival lissamine green staining scores (1.55 ± 1.19 to 0.36 ± 0.76 at three months, *p* = 0.001) compared to baseline [67].

The preoperative management of DED is important in ensuring accurate keratometry measurements, which can in turn influence intraocular lens (IOL) calculations [129]. Trattler and coworkers reported that among patients scheduled for cataract surgery, 77% of the eyes had evidence of corneal fluorescein staining, while 63% of patients had a measured TBUT of five seconds or less, although the proportion of symptomatic patients was not reported [130]. Tear film instability can affect keratometric measurements. A study of dry eye patients scheduled for cataract surgery reported that IOL calculations may vary by as much as 0.5D between measurements across different visits [131]. Epitropoulos and coworkers further reported that 8% of eyes with hyperosmolar tears had a difference of more than 0.50D in measured mean keratometry values across two visits (*p* = 0.049), and 17% of eyes had a vector astigmatism difference of more than 1.0D (*p* = 0.01). Similarly, Yang and coworkers evaluated the effect of DED on the reproducibility of keratometry measurements prior to cataract surgery and reported an inter-measurement difference of 0.28D in patients with DED versus 0.09D in healthy controls (*p* = 0.005) [132]. Hiraoka and coworkers conducted a study on patients scheduled for cataract surgery where keratometry readings were measured twice in the same day, ten minutes apart. Significant differences were observed in the keratometry readings of the steep meridian between repeated measurements, with a mean absolute difference of 0.21 ± 0.19D in the DED group and 0.14 ± 0.15D in the non-DED group (*p* = 0.044) [133]. These studies demonstrate larger differences in keratometric measurements among patients with DED. A multicentre prospective study of preoperative cataract surgery patients with DED found that the administration of DQS-LX significantly improved the ocular surface and reliability of keratometry measurements [68]. In this study, patients were diagnosed using the Japanese version of the OSDI (J-OSDI), underwent baseline biometry measurements and subsequently received DQS-LX thrice daily for four weeks. Post-treatment measurements demonstrated statistically significant improvements in TBUT (2.18 ± 0.80 s pre-treatment to 4.29 ± 1.14 s post-treatment, *p* < 0.001) and corneal higher-order aberrations (0.30 ± 0.03 µm pre-treatment to 0.25 ± 0.03 µm post-treatment, *p* < 0.001) after starting diquafosol. No statistically significant differences in measurements were seen in non-treated eyes across the measured time-points. Importantly, the durations between the last dose of DQS-LX and the keratometry measurements were not reported in the paper. Kobashi and coworkers have demonstrated improvements in mean intraocular scattering measured via the objective scattering index, a marker of optical quality, from 2.1 ± 0.7 before treatment to 1.5 ± 0.7 at two weeks (*p* < 0.001), and 1.6 ± 0.5 at four weeks (*p* < 0.001) after treatment, measured at least two hours after the last instillation of diquafosol [69]. The instillation of diquafosol has also been associated with an increase in LLT and TBUT measured at least 90 min following administration in support of these findings [46]. Further research on the duration of onset of tear film changes following diquafosol administration will be useful in the optimisation of keratometry measurements.

### 4.7. Contact Lens Wear

Contact lens wear compartmentalises the tear film into a pre- and post-lens tear film. This results in the thinning of the tear film, which contributes to its instability and increased friction between the contact lens and the ocular surface [134]. Contact lens wear has also been associated with meibomian gland dropout, which is postulated to be related to either a reduction in volume of the pre-lens tear film or a result of the direct mechanical effects of the contact lens [135]. These interactions can precipitate inflammation, which contributes to the further destabilisation of the tear film [134].

Diquafosol has been reported to create a statistically significant increase in post-instillation tear film volumes (*p* < 0.01 at 15 min and 30 min post-diquafosol instillation) in rabbit models of contact lens wear [136]. The topical application of diquafosol in soft contact lens users over a four-week treatment period resulted in a significant increase in the fluorescein intensities of the wheat germ agglutinin conjugate of fluorescein (F-WGA), which is used to quantify the presence of membrane-associated mucins (571.8 ± 227.8 µg/mL to 794.6 ± 219.4 µg/mL, *p* < 0.001), while stable sialic acid and tear protein concentrations suggest that diquafosol increased mucin secretion. This study also reported significant improvements in subjective symptoms measured by DEQS (19.2 ± 12.7 to 10.6 ± 12.5, *p* = 0.003), TBUT (3.6 ± 2.2 s to 5.0 ± 2.1 s, *p* = 0.003), conjunctival fluorescein staining score (2.8 ± 1.8 to 2.0 ± 1.4, *p* = 0.045) and corneal fluorescein staining score (0.7 ± 1.0 to 0.3 ± 0.6, *p* = 0.021) [70]. Another study involving soft contact lens wearers on either a daily or fortnightly replacement schedule reported that diquafosol significantly improved contrast sensitivity (*p* < 0.05) following eight weeks of usage six times daily. In this study, both corneal fluorescein staining and conjunctival lissamine green staining scores decreased markedly in the diquafosol group (*p* = 0.03 and *p* < 0.001, respectively), with significantly lower conjunctival lissamine green staining scores in the diquafosol group compared to soft contact lens wearers receiving artificial tears (*p* = 0.02). Among contact lens users, Ogami and coworkers reported more significant improvements with diquafosol instillation in symptoms of dryness and blurred vision (*p* < 0.01) compared to the administration of artificial tears. This was postulated to be due to both good compliance with diquafosol and its ability to promote the secretion of water and mucin, thereby stabilising the tear film [71].

Overnight orthokeratology lenses are an increasingly popular management modality for myopia control. However, 30–40% of patients experience ocular discomfort following lens insertion, which has been postulated to be due to tear film instability [137,138]. Similarly to soft contact lenses, rigid gas-permeable lenses can disrupt the tear film and induce meibomian gland atrophy [72,139]. A study by Xie and coworkers reported a significant increase in OSDI scores following three months of orthokeratology lens usage compared to baseline (baseline: 4.13 ± 4.21; third month: 7.26 ± 4.52, *p* < 0.05) [72]. A prospective study of paediatric orthokeratology lens wearers who were prescribed diquafosol four times daily for one month reported improved DEQS (5.54 ± 3.25 to 3.85 ± 2.98, *p* = 0.00), tear meniscus height (0.20 ± 0.05 mm to 0.21 ± 0.05 mm, *p* = 0.01) and TBUT (6.67 ± 4.71 s to 10.32 ± 6.19 s, *p* < 0.001) compared to baseline [73]. Thus, diquafosol may help to alleviate dry eye symptoms associated with paediatric orthokeratology lens wear and improve compliance, which is crucial for effective myopia management in children.

### 4.8. Keratorefractive Surgery

DED is the most common complication of keratorefractive surgery, with a reported prevalence of up to 75% of patients [140,141]. The pathogenesis of post-keratorefractive surgery dry eye is multifactorial, among which the surgical transection of sub-basal corneal nerves, which reduces the cornea blink reflex and disrupts the ocular surface–lacrimal gland neural loop, is a significant contributor [141]. This reduces the volume of tears secreted and induces tear film instability. Neurogenic inflammation, as evidenced by raised levels of pro-inflammatory tear mediators (IL-6, MMP-9), neuropeptides (Substance P and calcitonin gene-related peptide) and neuromediators (nerve growth factor), also contribute to post-keratorefractive surgery dry eye [142]. Structures of the ocular surface such as conjunctival goblet cells can inadvertently be damaged by surgical manipulation, such as during the application of suction devices in laser-assisted in situ keratomileusis (LASIK) and small-incision lenticule extraction (SMILE) [143]. Other factors include the administration of topical therapeutics, which may induce a toxic effect on the conjunctiva and cornea [144], as well as central corneal flattening, which affects lid–globe apposition with a resultant abnormal distribution of the tear film and adversely affected meibomian gland function [145].

A prospective study of DED patients who underwent SMILE reported that cornea and conjunctival fluorescein staining scores were lower in patients receiving diquafosol compared to sodium hyaluronate (1.20 ± 1.06 vs. 1.83 ± 1.41, respectively, *p* = 0.026) [74]. Additionally, the diquafosol group had better OSDI scores (12.98 ± 7.29 vs. 16.82 ± 8.25, *p* = 0.029), TBUTs (5.83 ± 2.02 s vs. 4.24 ± 0.94 s, *p* < 0.001) and Schirmer’s test scores (7.75 ± 3.92 mm vs. 5.24 ± 3.42 mm, *p* = 0.003) three months post-surgery. A prospective study investigated the efficacy of diquafosol in treating patients experiencing persistent dry eye symptoms for over 12 months following LASIK [75]. The study compared the effects of diquafosol with those of artificial tears over a 12-week period. Results demonstrated that diquafosol significantly improved both subjective and objective parameters. Although the results of Schirmer’s test were not significantly different after the addition of diquafosol (7.4 ± 5.1 mm pre-treatment vs. 6.8 ± 3.65 mm at 12 weeks post-treatment, *p* = 0.48), TBUT improved at one-week (*p* = 0.007), 4 weeks (*p* = 0.001) and 12 weeks (*p* < 0.001) compared to baseline. However, the exact improvement in TBUT was not reported. Corneal fluorescein and conjunctival lissamine green staining scores improved within one week of initiating diquafosol treatment (*p* < 0.001). Reported subjective symptoms, measured using a modified OSDI tool, demonstrated reductions in fatigue, discomfort, dryness, grittiness and difficulty reading (*p* < 0.05).

In patients undergoing femtosecond LASIK (FS-LASIK), regardless of whether dry eye was present, combining diquafosol six times a day and sodium hyaluronate four times a day postoperatively for a month significantly improved postoperative subjective symptoms, ocular surface status and LLT compared to monotherapy with sodium hyaluronate [76]. Additionally, all patients also received routine post-procedural eyedrops, such as antibiotics and anti-inflammatory agents. The use of combination therapy significantly lowered OSDI scores (diquafosol and sodium hyaluronate: 17.55 ± 15.70 to 16.97 ± 9.96; sodium hyaluronate: 18.39 ± 17.31 to 28.72 ± 19.65; *p* = 0.024) following FS-LASIK at one-month postoperation. While there was no significant difference in corneal fluorescein staining score for the combination group between preoperative and postoperative visits, score increments were significantly lower for the combination group compared to the sodium hyaluronate group at one week (*p* = 0.018) and not statistically significant at one month after FS-LASIK. Furthermore, in patients without preoperative dry eye symptoms, the addition of diquafosol resulted in better-retained corneal sensitivity, measured with a Cochet–Bonnet esthesiometer one month after FS-LASIK, compared to patients who only used sodium hyaluronate (26.43  ±  20.80 mm vs. 12.237  ±  14.86 mm, *p* = 0.041). The confocal microscopic analysis of DED patients has suggested a possible role of diquafosol (administrated six times daily for three months) in increasing sub-basal corneal nerve density (baseline: 829.6 ± 348.0 μm/mm^2^; three months: 1238.6 ± 410.1 μm/mm^2^, *p* = 0.02) [77].

### 4.9. Long-Acting Diquafosol (DQS-LX) Formulation

Diquafosol is usually prescribed up to six times daily for maximal efficacy. Compliance, however, is challenging, with only 10.2% of participants reported to be compliant with the recommended administration frequency in a study [146]. A new long-acting diquafosol formulation which is administered thrice daily has recently been developed via the addition of polyvinylpyrrolidone (PVP). It confers several benefits, including increased viscosity and higher levels of lipid secretion, more effectively compared to standard diquafosol formulations [80]. It is postulated that its increased viscosity reduces ocular surface irritation, improves tear fluid stability and reduces corneal and conjunctival epithelial damage. The lipid-increasing effect on the tear film may further minimise the friction generated during lid–globe interactions and reduce evaporative loss. DQS-LX is dosed at three times daily, as studies have shown that the effects of this dose on corneal fluorescein staining score and TBUT were similar to when conventional diquafosol was used six times daily [78].

A randomised controlled trial of patients with aqueous-deficient DED receiving DQS-LX were reported to experience improvements in corneal fluorescein staining (MD: −0.51; *p* < 0.0001) and conjunctival lissamine green staining scores (MD: −0.33; *p* = 0.0093) after four weeks of administration compared the vehicle control group [78]. The common adverse symptoms reported were eye irritation (3.6%) and discharge (1.8%) of mild severity.

Another study by Ishikawa and coworkers investigating the compliance rates of patients using diquafosol reported that switching to a long-acting formulation improved adherence rates from 5.6%, among those prescribed a conventional diquafosol formulation six times daily, to 88.9% in patients prescribed DQS-LX to be administered three times daily [79]. In this study, both formulations were found to be equally effective in improving TBUT.

Patients with reduced tear film volume or moderate–severe dry eyes with meibomian gland dysfunction have reported preferences for the long-acting formulation, while those with allergic findings such as conjunctival papillae expressed their preference for conventional diquafosol [80]. The study cited eye stickiness, discharge, itchiness and irritation as reasons for reverting to conventional diquafosol. Notably, patients with papillary and follicular changes in the conjunctiva are more symptomatic regardless of the severity of dry eye symptoms [147]. The preference for conventional diquafosol may be attributable to PVP, which increases tear viscosity and prolongs allergen residence time on the ocular surface, exacerbating itch. Additionally, patients with allergic conjunctivitis have been described to experience impaired blink mechanisms, which may affect the lacrimal pump mechanism and contribute to tear retention [148]. Likely explanations for this include changes in the palpebral conjunctival anatomy due to papillae formation, as well as persistent itching, which can disrupt normal blinking patterns.

## 5. Conclusions

Existing evidence suggests that diquafosol is a useful addition to our treatment armamentarium in managing a range of ocular surface conditions, though the exact mechanisms underpinning its reported range of benefits have yet to be fully elucidated. Clinicians considering prescribing diquafosol to their patients should be aware of its potential side effects, particularly when initiating therapy. Future efforts ought to focus on conducting rigorous high-quality trials to evaluate the effectiveness of diquafosol across the spectrum of ocular surface diseases against and alongside other pharmacological and office-based interventions, and the effects of the long-term cessation of diquafosol, to optimise treatment outcomes for patients with ocular surface disorders.

## Figures and Tables

**Table 1:** Composition of Diquas^®^, Diquas^®^-S and Diquas^®^-LX (long-acting).

Diquas^®^	Diquas^®^-S	Diquas^®^-LX
Diquafosol sodium 3% Chlorhexidine gluconate (preservative) Dibasic sodium phosphate hydrate Disodium edetate hydrate Sodium chloride Potassium chloride Sodium hydroxide Dilute hydrochloric acid	Diquafosol sodium 3% Dibasic sodium phosphate hydrate Disodium edetate hydrate Sodium chloride Potassium chloride Hydrochloric acid Sodium hydroxide	Diquafosol sodium 3% Dibasic sodium phosphate hydrate Disodium edetate hydrate Sodium chloride Polyvinylpyrrolidone Silver nitrate pH adjuster

**Table 2:** Reported adverse effects of topical diquafosol 3%.

Ophthalmic Adverse Effects
Ocular irritation
Ocular discharge
Foreign body sensation
Conjunctival hyperaemia
Ocular pain and discomfort
Ocular pruritus

**Table 3:** Summary of study conclusions.

Condition	Study Type	Summary of Study Conclusions	References
Dry Eye Disease	Diquafosol 3% vs. artificial tears	Diquafosol improved the following: dry eye symptoms corneal fluorescein staining score conjunctival rose bengal staining score tear break-up time Schirmer’s test	[16,24,25,26,30,53,54]
	Diquafosol 3% vs. cyclosporine	Diquafosol 3% vs. cyclosporine 0.05% vs. cyclosporine 0.1% showed the following: all three medications downregulated tear proteomes all three medications improved corneal fluoresceine staining score and tear break-up time corneal fluorescein staining score improved more significantly among cyclosporine users Combination therapy of diquafosol 3% and cyclosporine 0.1% vs. cyclosporine 0.1% demonstrated the following: combination therapy provided greater improvement in tear break-up time	[55,56]
Meibomian Gland Dysfunction	Pre- vs. post-diquafosol 3% instillation	Diquafosol improved the following: dry eye symptoms meibomian gland area lid margin abnormalities	[45,57]
	Diquafosol 3% vs. artificial tears vs. gatifloxacin 0.3%	Only diquafosol increased lipid layer thickness	[58]
Aqueous-Deficient Dry Eye Disease	Pre- vs. post-diquafosol 3% instillation	Diquafosol improved the following: dry eye symptoms corneal and conjunctival fluorescein staining score tear break-up time tear volume optical quality of tear film	[42,59,60,61]
Ocular Graft-Versus-Host Disease	Pre- vs. post-diquafosol 3% instillation	Diquafosol improved the following: dry eye symptoms corneal fluorescein staining score corneal and conjunctival rose bengal staining score tear break-up time	[62,63]
Glaucoma Medication- and Preservative-Related Ocular Surface Disease	Pre- vs. post-diquafosol 3% instillation	Diquafosol improved: dry eye symptoms tear break-up time schirmer’s test goblet cell density tear meniscus height after trabeculectomy lipid layer thickness after trabeculectomy Diquafosol also demostrated a protective effect against meibomian gland dropout among patients using prostaglandin analogue.	[64,65,66]
Cataract Surgery	Diquafosol 3% vs. artificial tears	Postoperatively, diquafosol improved the following: dry eye symptoms corneal fluorescein staining score conjunctival lissamine green staining score tear break-up time	[21,67]
	Pre- vs. post-diquafosol 3% instillation	Pre-keratometry administration of diquafosol improved the following: tear break-up time accuracy and reliability of keratometry measurements	[68,69]
Contact Lens Wear	Pre- vs. post-diquafosol 3% instillation Diquafosol 3% vs. artificial tears	Diquafosol improved the following: dry eye symptoms tear break-up time corneal and conjunctival fluorescein staining score conjunctival lissamine green staining score Among paediatric orthokeratology lens users, diquafosol improved the following: dry eye symptoms tear meniscus height	[70,71,72,73]
Keratorefractive Surgery	Diquafosol 3% vs. artificial tears	Among patients who underwent small-incision lenticule extraction (SMILE), diquafosol improved the following: dry eye symptoms conjunctival fluorescein staining score Schirmer’s test Among patients who underwent laser-assisted in situ keratomileusis (LASIK), diquafosol improved the following: dry eye symptoms tear break-up time corneal fluorescein staining score conjunctival lissamine green staining score corneal sensitivity and sub-basal corneal nerve density	[74,75,76,77]
Long-Acting Diquafosol	Long-acting diquafosol 3% vs. vehicle	Long-acting diquafosol improved the following: corneal fluorescein staining score conjunctival lissamine green staining score	[78]
	Long-acting diquafosol 3% vs. conventional diquafosol 3%	Long-acting formulation, compared to conventional diquafosol, provided better patient compliance	[79,80]

## References

[1] Wu D., Tong L., Prasath A., Lim B.X.H., Lim D.K., Lim C.H.L. (2023). Novel therapeutics for dry eye disease. Ann. Med..

[2] Cai Y., Wei J., Zhou J., Zou W. (2022). Prevalence and Incidence of Dry Eye Disease in Asia: A Systematic Review and Meta-Analysis. Ophthalmic Res..

[3] Song P., Xia W., Wang M., Chang X., Wang J., Jin S., Wang J., Wei W., Rudan I. (2018). Variations of dry eye disease prevalence by age, sex and geographic characteristics in China: A systematic review and meta-analysis. J. Glob. Health.

[4] Uchino M., Schaumberg D.A. (2013). Dry Eye Disease: Impact on Quality of Life and Vision. Curr. Ophthalmol. Rep..

[5] Buchholz P., Steeds C.S., Stern L.S., Wiederkehr D.P., Doyle J.J., Katz L.M., Figueiredo F.C. (2006). Utility assessment to measure the impact of dry eye disease. Ocul. Surf..

[6] Yang W., Luo Y., Wu S., Niu X., Yan Y., Qiao C., Ming W., Zhang Y., Wang H., Chen D. (2021). Estimated Annual Economic Burden of Dry Eye Disease Based on a Multi-Center Analysis in China: A Retrospective Study. Front. Med..

[7] Yu J., Asche C.V., Fairchild C.J. (2011). The economic burden of dry eye disease in the United States: A decision tree analysis. Cornea.

[8] Craig J.P., Nichols K.K., Akpek E.K., Caffery B., Dua H.S., Joo C.K., Liu Z., Nelson J.D., Nichols J.J., Tsubota K. (2017). TFOS DEWS II Definition and Classification Report. Ocul. Surf..

[9] Tsubota K., Yokoi N., Watanabe H., Dogru M., Kojima T., Yamada M., Kinoshita S., Kim H.M., Tchah H.W., Hyon J.Y. (2020). A New Perspective on Dry Eye Classification: Proposal by the Asia Dry Eye Society. Eye Contact Lens.

[10] Yokoi N., Georgiev G.A. (2018). Tear Film-Oriented Diagnosis and Tear Film-Oriented Therapy for Dry Eye Based on Tear Film Dynamics. Investig. Ophthalmol. Vis. Sci..

[11] Barbosa Ribeiro B., Marta A., Ponces Ramalhão J., Marques J.H., Barbosa I. (2022). Pulsed Light Therapy in the Management of Dry Eye Disease: Current Perspectives. Clin. Ophthalmol..

[12] Blades K.J., Patel S., Aidoo K.E. (2001). Oral antioxidant therapy for marginal dry eye. Eur. J. Clin. Nutr..

[13] Chan K.E., Lau B.S.R., Lim B.X.H., Du R., Giannaccare G., Tong L., Stapleton F., Lim C.H.L. (2024). Low-level light therapy and intense pulse light therapy in meibomian gland dysfunction. A systematic review and meta-analysis. Contact Lens Anterior Eye.

[14] Nakamura M., Imanaka T., Sakamoto A. (2012). Diquafosol ophthalmic solution for dry eye treatment. Adv. Ther..

[15] Kojima T., Nagata T., Kudo H., Müller-Lierheim W.G.K., van Setten G.B., Dogru M., Tsubota K. (2020). The Effects of High Molecular Weight Hyaluronic Acid Eye Drop Application in Environmental Dry Eye Stress Model Mice. Int. J. Mol. Sci..

[16] Sun X., Liu L., Liu C. (2023). Topical diquafosol versus hyaluronic acid for the treatment of dry eye disease: A meta-analysis of randomized controlled trials. Graefes Arch. Clin. Exp. Ophthalmol..

[17] Cowlen M.S., Zhang V.Z., Warnock L., Moyer C.F., Peterson W.M., Yerxa B.R. (2003). Localization of ocular P2Y2 receptor gene expression by in situ hybridization. Exp. Eye Res..

[18] Murakami T., Fujihara T., Horibe Y., Nakamura M. (2004). Diquafosol elicits increases in net Cl- transport through P2Y2 receptor stimulation in rabbit conjunctiva. Ophthalmic Res..

[19] Lee H.J., Yang S., Cheon E.J., Shin S., Byun Y.S., Kim H.S., Chung S.H. (2022). Diquafosol ophthalmic solution enhances mucin expression via ERK activation in human conjunctival epithelial cells with hyperosmotic stress. Mol. Vis..

[20] (2010). Report on the Deliberation Results. https://www.pmda.go.jp/files/000153954.pdf.

[21] Jun I., Choi S., Lee G.Y., Choi Y.J., Lee H.K., Kim E.K., Seo K.Y., Kim T.I. (2019). Effects of Preservative-free 3% Diquafosol in Patients with Pre-existing Dry Eye Disease after Cataract Surgery: A Randomized Clinical Trial. Sci. Rep..

[22] Inspire Announces Results of Phase 3 PROLACRIA Trial for Dry Eye. Fierce Biotech. https://www.fiercebiotech.com/biotech/inspire-announces-results-of-phase-3-prolacria-trial-for-dry-eye.

[23] Matsumoto Y., Ohashi Y., Watanabe H., Tsubota K. (2012). Efficacy and safety of diquafosol ophthalmic solution in patients with dry eye syndrome: A Japanese phase 2 clinical trial. Ophthalmology.

[24] Takamura E., Tsubota K., Watanabe H., Ohashi Y. (2012). A randomised, double-masked comparison study of diquafosol versus sodium hyaluronate ophthalmic solutions in dry eye patients. Br. J. Ophthalmol..

[25] Tauber J., Davitt W.F., Bokosky J.E., Nichols K.K., Yerxa B.R., Schaberg A.E., LaVange L.M., Mills-Wilson M.C., Kellerman D.J. (2004). Double-masked, placebo-controlled safety and efficacy trial of diquafosol tetrasodium (INS365) ophthalmic solution for the treatment of dry eye. Cornea.

[26] Gong L., Sun X., Ma Z., Wang Q., Xu X., Chen X., Shao Y., Yao K., Tang L., Gu Y. (2015). A randomised, parallel-group comparison study of diquafosol ophthalmic solution in patients with dry eye in China and Singapore. Br. J. Ophthalmol..

[27] Kamiya K., Nakanishi M., Ishii R., Kobashi H., Igarashi A., Sato N., Shimizu K. (2012). Clinical evaluation of the additive effect of diquafosol tetrasodium on sodium hyaluronate monotherapy in patients with dry eye syndrome: A prospective, randomized, multicenter study. Eye.

[28] Hwang H.S., Sung Y.M., Lee W.S., Kim E.C. (2014). Additive Effect of preservative-free sodium hyaluronate 0.1% in treatment of dry eye syndrome with diquafosol 3% eye drops. Cornea.

[29] Shimazaki-Den S., Iseda H., Dogru M., Shimazaki J. (2013). Effects of diquafosol sodium eye drops on tear film stability in short BUT type of dry eye. Cornea.

[30] Ohashi Y., Munesue M., Shimazaki J., Takamura E., Yokoi N., Watanabe H., Nomura A., Shimada F. (2020). Long-Term Safety and Effectiveness of Diquafosol for the Treatment of Dry Eye in a Real-World Setting: A Prospective Observational Study. Adv. Ther..

[31] Idzko M., Ferrari D., Eltzschig H.K. (2014). Nucleotide signalling during inflammation. Nature.

[32] Peterson T.S., Camden J.M., Wang Y., Seye C.I., Wood W.G., Sun G.Y., Erb L., Petris M.J., Weisman G.A. (2010). P2Y2 nucleotide receptor-mediated responses in brain cells. Mol. Neurobiol..

[33] Kargarpour Z., Cicko S., Köhler T.C., Zech A., Stoshikj S., Bal C., Renner A., Idzko M., El-Gazzar A. (2023). Blocking P2Y2 purinergic receptor prevents the development of lipopolysaccharide-induced acute respiratory distress syndrome. Front. Immunol..

[34] Bellefeuille S.D., Molle C.M., Gendron F.P. (2019). Reviewing the role of P2Y receptors in specific gastrointestinal cancers. Purinergic Signal..

[35] Elliott M.R., Chekeni F.B., Trampont P.C., Lazarowski E.R., Kadl A., Walk S.F., Park D., Woodson R.I., Ostankovich M., Sharma P. (2009). Nucleotides released by apoptotic cells act as a find-me signal to promote phagocytic clearance. Nature.

[36] Bremond-Gignac D., Gicquel J.J., Chiambaretta F. (2014). Pharmacokinetic evaluation of diquafosol tetrasodium for the treatment of Sjögren’s syndrome. Expert Opin. Drug Metab. Toxicol..

[37] Dota A., Sakamoto A., Nagano T., Murakami T., Matsugi T. (2020). Effect of Diquafosol Ophthalmic Solution on Airflow-Induced Ocular Surface Disorder in Diabetic Rats. Clin. Ophthalmol..

[38] Byun Y.S., Yoo Y.S., Kwon J.Y., Joo J.S., Lim S.A., Whang W.J., Mok J.W., Choi J.S., Joo C.K. (2016). Diquafosol promotes corneal epithelial healing via intracellular calcium-mediated ERK activation. Exp. Eye Res..

[39] Yokoi N., Kato H., Kinoshita S. (2014). Facilitation of tear fluid secretion by 3% diquafosol ophthalmic solution in normal human eyes. Am. J. Ophthalmol..

[40] Oguz H., Yokoi N., Kinoshita S. (2000). The height and radius of the tear meniscus and methods for examining these parameters. Cornea.

[41] Yokoi N., Bron A.J., Tiffany J.M., Kinoshita S. (2000). Reflective meniscometry: A new field of dry eye assessment. Cornea.

[42] Yokoi N., Kato H., Kinoshita S. (2016). The increase of aqueous tear volume by diquafosol sodium in dry-eye patients with Sjögren’s syndrome: A pilot study. Eye.

[43] Ikeda K., Simsek C., Kojima T., Higa K., Kawashima M., Dogru M., Shimizu T., Tsubota K., Shimazaki J. (2018). The effects of 3% diquafosol sodium eye drop application on meibomian gland and ocular surface alterations in the Cu, Zn-superoxide dismutase-1 (Sod1) knockout mice. Graefes Arch. Clin. Exp. Ophthalmol..

[44] Endo K.I., Sakamoto A., Fujisawa K. (2021). Diquafosol tetrasodium elicits total cholesterol release from rabbit meibomian gland cells via P2Y(2) purinergic receptor signalling. Sci. Rep..

[45] Fukuoka S., Arita R. (2017). Increase in tear film lipid layer thickness after instillation of 3% diquafosol ophthalmic solution in healthy human eyes. Ocul. Surf..

[46] Fukuoka S., Arita R. (2019). Tear film lipid layer increase after diquafosol instillation in dry eye patients with meibomian gland dysfunction: A randomized clinical study. Sci. Rep..

[47] Inatomi T., Spurr-Michaud S., Tisdale A.S., Zhan Q., Feldman S.T., Gipson I.K. (1996). Expression of secretory mucin genes by human conjunctival epithelia. Investig. Ophthalmol. Vis. Sci..

[48] Gipson I.K. (2004). Distribution of mucins at the ocular surface. Exp. Eye Res..

[49] Mantelli F., Argüeso P. (2008). Functions of ocular surface mucins in health and disease. Curr. Opin. Allergy Clin. Immunol..

[50] Jin Y., Seo K.Y., Kim S.W. (2024). Comparing two mucin secretagogues for the treatment of dry eye disease: A prospective randomized crossover trial. Sci. Rep..

[51] Hori Y., Kageyama T., Sakamoto A., Shiba T., Nakamura M., Maeno T. (2017). Comparison of Short-Term Effects of Diquafosol and Rebamipide on Mucin 5AC Level on the Rabbit Ocular Surface. J. Ocul. Pharmacol. Ther..

[52] Terakado K., Yogo T., Kohara Y., Soeta S., Nezu Y., Harada Y., Hara Y., Amasaki H., Tagawa M. (2014). Conjunctival expression of the P2Y2 receptor and the effects of 3% diquafosol ophthalmic solution in dogs. Vet. J..

[53] Liu S., Yang G., Li Q., Tang S. (2023). Safety and efficacy of topical diquafosol for the treatment of dry eye disease: An updated meta-analysis of randomized controlled trials. Indian J. Ophthalmol..

[54] Nam K.T., Ahn S.M., Eom Y., Kim H.M., Song J.S. (2015). Immediate Effects of 3% Diquafosol and 0.1% Hyaluronic Acid Ophthalmic Solution on Tear Break-Up Time in Normal Human Eyes. J. Ocul. Pharmacol. Ther..

[55] Jung G.T., Kim M., Song J.S., Kim T.I., Chung T.Y., Choi C.Y., Kim H.S., An W.J., Jeong S.J., Lee H.S. (2023). Proteomic analysis of tears in dry eye disease: A prospective, double-blind multicenter study. Ocul. Surf..

[56] Eom Y., Song J.S., Kim H.M. (2022). Effectiveness of Topical Cyclosporin A 0.1%, Diquafosol Tetrasodium 3%, and Their Combination, in Dry Eye Disease. J. Ocul. Pharmacol. Ther..

[57] Arita R., Suehiro J., Haraguchi T., Maeda S., Maeda K., Tokoro H., Amano S. (2013). Topical diquafosol for patients with obstructive meibomian gland dysfunction. Br. J. Ophthalmol..

[58] Kang D.H., Lee Y.W., Hwang K.Y., Koh K.M., Kwon Y.A., Kim B.Y., Song S.W., Kim K.Y. (2019). Changes of tear film lipid layer thickness by 3% diquafosol ophthalmic solutions in patients with dry eye syndrome. Int. J. Ophthalmol..

[59] Koh S., Ikeda C., Takai Y., Watanabe H., Maeda N., Nishida K. (2013). Long-term results of treatment with diquafosol ophthalmic solution for aqueous-deficient dry eye. Jpn. J. Ophthalmol..

[60] Koh S., Maeda N., Ikeda C., Oie Y., Soma T., Tsujikawa M., Watanabe H., Nishida K. (2014). Effect of diquafosol ophthalmic solution on the optical quality of the eyes in patients with aqueous-deficient dry eye. Acta Ophthalmol..

[61] Yokoi N., Sonomura Y., Kato H., Komuro A., Kinoshita S. (2015). Three percent diquafosol ophthalmic solution as an additional therapy to existing artificial tears with steroids for dry-eye patients with Sjögren’s syndrome. Eye.

[62] Yamane M., Ogawa Y., Fukui M., Kamoi M., Uchino M., Saijo-Ban Y., Kozuki N., Mukai S., Mori T., Okamoto S. (2018). Long-Term Topical Diquafosol Tetrasodium Treatment of Dry Eye Disease Caused by Chronic Graft-Versus-Host Disease: A Retrospective Study. Eye Contact Lens.

[63] Yamane M., Ogawa Y., Fukui M., Kamoi M., Saijo-Ban Y., Yaguchi S., Mukai S., Kawakita T., Simmura S., Tsubota K. (2015). Long-term rebamipide and diquafosol in two cases of immune-mediated dry eye. Optom. Vis. Sci..

[64] Jin S.W., Min J.S. (2016). Clinical evaluation of the effect of diquafosol ophthalmic solution in glaucoma patients with dry eye syndrome. Jpn. J. Ophthalmol..

[65] Guo Y., Ha J.Y., Piao H.L., Sung M.S., Park S.W. (2020). The protective effect of 3% diquafosol on meibomian gland morphology in glaucoma patients treated with prostaglandin analogs: A 12-month follow-up study. BMC Ophthalmol..

[66] Liu Q., Cheng W., Liu C., Jin X., Ming S., Zhao D., Feng X. (2023). Evaluation of effects of 3% diquafosol ophthalmic solution on preocular tear film stability after trabeculectomy. Int. Ophthalmol..

[67] Park D.H., Chung J.K., Seo D.R., Lee S.J. (2016). Clinical Effects and Safety of 3% Diquafosol Ophthalmic Solution for Patients with Dry Eye After Cataract Surgery: A Randomized Controlled Trial. Am. J. Ophthalmol..

[68] Teshigawara T., Akaishi M., Mizuki Y., Takeuchi M., Hata S., Meguro A., Mizuki N. (2024). Effect of Long-Acting Diquafosol Sodium on Astigmatism Measurement Repeatability in Preoperative Cataract Cases with Dry Eyes: A Multicenter Prospective Study. Ophthalmol. Ther..

[69] Kobashi H., Kamiya K., Igarashi A., Miyake T., Shimizu K. (2015). Intraocular Scattering after Instillation of Diquafosol Ophthalmic Solution. Optom. Vis. Sci..

[70] Shigeyasu C., Yamada M., Akune Y., Fukui M. (2016). Diquafosol for Soft Contact Lens Dryness: Clinical Evaluation and Tear Analysis. Optom. Vis. Sci..

[71] Ogami T., Asano H., Hiraoka T., Yamada Y., Oshika T. (2021). The Effect of Diquafosol Ophthalmic Solution on Clinical Parameters and Visual Function in Soft Contact Lens-Related Dry Eye. Adv. Ther..

[72] Xie C., Wei R. (2023). Long-term changes in the ocular surface during orthokeratology lens wear and their correlations with ocular discomfort symptoms. Contact Lens Anterior Eye.

[73] Yang Y., Wu Q., Tang Y., Wu H., Luo Z., Gao W., Hu Z., Hou L., Wang M., Yang Z. (2023). Short-term application of diquafosol ophthalmic solution benefits children with dry eye wearing orthokeratology lens. Front. Med..

[74] Liu Y., Qian Y., Li M., Shi Y., Liu L., Sun L., Ye H., Zou J. (2023). Combination Therapy with Diquafosol Sodium and Sodium Hyaluronate in Eyes with Dry Eye Disease After Small Incision Lenticule Extraction. In Vivo.

[75] Mori Y., Nejima R., Masuda A., Maruyama Y., Minami K., Miyata K., Amano S. (2014). Effect of diquafosol tetrasodium eye drop for persistent dry eye after laser in situ keratomileusis. Cornea.

[76] Wang T., Di Y., Li Y. (2023). Combination therapy with 3% diquafosol tetrasodium ophthalmic solution and sodium hyaluronate: An effective therapy for patients with dry eye after femtosecond laser-assisted in situ keratomileusis. Front. Med..

[77] Matsumoto Y., Ibrahim O.M.A., Kojima T., Dogru M., Shimazaki J., Tsubota K. (2020). Corneal In Vivo Laser-Scanning Confocal Microscopy Findings in Dry Eye Patients with Sjögren’s Syndrome. Diagnostics.

[78] Hori Y., Oka K., Inai M. (2022). Efficacy and Safety of the Long-Acting Diquafosol Ophthalmic Solution DE-089C in Patients with Dry Eye: A Randomized, Double-Masked, Placebo-Controlled Phase 3 Study. Adv. Ther..

[79] Ishikawa S., Sasaki T., Maruyama T., Murayama K., Shinoda K. (2023). Effectiveness and Adherence of Dry Eye Patients Who Switched from Short- to Long-Acting Diquafosol Ophthalmic Solution. J. Clin. Med..

[80] Arita R., Fukuoka S., Kaido M. (2024). Tolerability of Diquas LX on tear film and meibomian glands findings in a real clinical scenario. PLoS ONE.

[81] Kim Y.H., Yang I.J., Nguyen L.T.H., Gum S.I., Yu S., Lee G.J., Kim B.A., Jung J.C., Park Y.J. (2020). Effect of Diquafosol on Hyperosmotic Stress-induced Tumor Necrosis Factor-α and Interleukin-6 Expression in Human Corneal Epithelial Cells. Korean J. Ophthalmol..

[82] Ozdemir S., Yeo S.W.J., Lee J.J., Bhaskar A., Finkelstein E., Tong L. (2022). Patient Medication Preferences for Managing Dry Eye Disease: The Importance of Medication Side Effects. Patient.

[83] Sheppard J.D., Nichols K.K. (2023). Dry Eye Disease Associated with Meibomian Gland Dysfunction: Focus on Tear Film Characteristics and the Therapeutic Landscape. Ophthalmol. Ther..

[84] Donthineni P.R., Kammari P., Shanbhag S.S., Singh V., Das A.V., Basu S. (2019). Incidence, demographics, types and risk factors of dry eye disease in India: Electronic medical records driven big data analytics report I. Ocul. Surf..

[85] Donthineni P.R., Doctor M.B., Shanbhag S., Kate A., Galor A., Djalilian A.R., Singh S., Basu S. (2023). Aqueous-deficient dry eye disease: Preferred practice pattern guidelines on clinical approach, diagnosis, and management. Indian J. Ophthalmol..

[86] Malard F., Holler E., Sandmaier B.M., Huang H., Mohty M. (2023). Acute graft-versus-host disease. Nat. Rev. Dis. Primers.

[87] Sakoda Y., Hashimoto D., Asakura S., Takeuchi K., Harada M., Tanimoto M., Teshima T. (2007). Donor-derived thymic-dependent T cells cause chronic graft-versus-host disease. Blood.

[88] Surico P.L., Luo Z.K. (2024). Understanding Ocular Graft-versus-Host Disease to Facilitate an Integrated Multidisciplinary Approach. Transplant. Cell. Ther..

[89] Yang F., Hayashi I., Sato S., Saijo-Ban Y., Yamane M., Fukui M., Shimizu E., He J., Shibata S., Mukai S. (2022). Eyelid blood vessel and meibomian gland changes in a sclerodermatous chronic GVHD mouse model. Ocul. Surf..

[90] Perez V.L., Mousa H.M., Soifer M., Beatty C., Sarantopoulos S., Saban D.R., Levy R.B. (2023). Meibomian Gland Dysfunction: A Route of Ocular Graft-Versus-Host Disease Progression That Drives a Vicious Cycle of Ocular Surface Inflammatory Damage. Am. J. Ophthalmol..

[91] Ogawa Y., Shimmura S., Kawakita T., Yoshida S., Kawakami Y., Tsubota K. (2009). Epithelial mesenchymal transition in human ocular chronic graft-versus-host disease. Am. J. Pathol..

[92] Shamloo K., Barbarino A., Alfuraih S., Sharma A. (2019). Graft Versus Host Disease-Associated Dry Eye: Role of Ocular Surface Mucins and the Effect of Rebamipide, a Mucin Secretagogue. Investig. Ophthalmol. Vis. Sci..

[93] Kolko M., Gazzard G., Baudouin C., Beier S., Brignole-Baudouin F., Cvenkel B., Fineide F., Hedengran A., Hommer A., Jespersen E. (2023). Impact of glaucoma medications on the ocular surface and how ocular surface disease can influence glaucoma treatment. Ocul. Surf..

[94] Zhang X., Vadoothker S., Munir W.M., Saeedi O. (2019). Ocular Surface Disease and Glaucoma Medications: A Clinical Approach. Eye Contact Lens.

[95] Coakes R.L., Mackie I.A., Seal D.V. (1981). Effects of long-term treatment with timolol on lacrimal gland function. Br. J. Ophthalmol..

[96] Kuppens E.V., de Jong C.A., Stolwijk T.R., de Keizer R.J., van Best J.A. (1995). Effect of timolol with and without preservative on the basal tear turnover in glaucoma. Br. J. Ophthalmol..

[97] Zhang Y., Kam W.R., Liu Y., Chen X., Sullivan D.A. (2017). Influence of Pilocarpine and Timolol on Human Meibomian Gland Epithelial Cells. Cornea.

[98] Aydin Kurna S., Acikgoz S., Altun A., Ozbay N., Sengor T., Olcaysu O.O. (2014). The effects of topical antiglaucoma drugs as monotherapy on the ocular surface: A prospective study. J. Ophthalmol..

[99] Nijm L.M., De Benito-Llopis L., Rossi G.C., Vajaranant T.S., Coroneo M.T. (2020). Understanding the Dual Dilemma of Dry Eye and Glaucoma: An International Review. Asia Pac. J. Ophthalmol..

[100] Mocan M.C., Uzunosmanoglu E., Kocabeyoglu S., Karakaya J., Irkec M. (2016). The Association of Chronic Topical Prostaglandin Analog Use with Meibomian Gland Dysfunction. J. Glaucoma.

[101] Rabinowitz M.P., Katz L.J., Moster M.R., Myers J.S., Pro M.J., Spaeth G.L., Sharma P., Stefanyszyn M.A. (2015). Unilateral Prostaglandin-Associated Periorbitopathy: A Syndrome Involving Upper Eyelid Retraction Distinguishable from the Aging Sunken Eyelid. Ophthalmic Plast. Reconstr. Surg..

[102] Vitoux M.A., Kessal K., Melik Parsadaniantz S., Claret M., Guerin C., Baudouin C., Brignole-Baudouin F., Réaux-Le Goazigo A. (2020). Benzalkonium chloride-induced direct and indirect toxicity on corneal epithelial and trigeminal neuronal cells: Proinflammatory and apoptotic responses in vitro. Toxicol. Lett..

[103] Ivakhnitskaia E., Souboch V., Dallacasagrande V., Mizerska K., Souboch E., Sarkar J., Guaiquil V.H., Tseng K.Y., Hirata H., Rosenblatt M.I. (2022). Benzalkonium chloride, a common ophthalmic preservative, compromises rat corneal cold sensitive nerve activity. Ocul. Surf..

[104] Van Went C., Alalwani H., Brasnu E., Pham J., Hamard P., Baudouin C., Labbé A. (2011). Corneal sensitivity in patients treated medically for glaucoma or ocular hypertension. J. Fr. Ophtalmol..

[105] Martone G., Frezzotti P., Tosi G.M., Traversi C., Mittica V., Malandrini A., Pichierri P., Balestrazzi A., Motolese P.A., Motolese I. (2009). An in vivo confocal microscopy analysis of effects of topical antiglaucoma therapy with preservative on corneal innervation and morphology. Am. J. Ophthalmol..

[106] Ammar D.A., Noecker R.J., Kahook M.Y. (2010). Effects of benzalkonium chloride-preserved, polyquad-preserved, and sofZia-preserved topical glaucoma medications on human ocular epithelial cells. Adv. Ther..

[107] Liang H., Brignole-Baudouin F., Riancho L., Baudouin C. (2012). Reduced in vivo ocular surface toxicity with polyquad-preserved travoprost versus benzalkonium-preserved travoprost or latanoprost ophthalmic solutions. Ophthalmic Res..

[108] Kahook M.Y., Rapuano C.J., Messmer E.M., Radcliffe N.M., Galor A., Baudouin C. (2024). Preservatives and ocular surface disease: A review. Ocul. Surf..

[109] Herreras J.M., Pastor J.C., Calonge M., Asensio V.M. (1992). Ocular surface alteration after long-term treatment with an antiglaucomatous drug. Ophthalmology.

[110] Chung S.H., Lee S.K., Cristol S.M., Lee E.S., Lee D.W., Seo K.Y., Kim E.K. (2006). Impact of short-term exposure of commercial eyedrops preserved with benzalkonium chloride on precorneal mucin. Mol. Vis..

[111] Martinez-de-la-Casa J.M., Perez-Bartolome F., Urcelay E., Santiago J.L., Moreno-Montañes J., Arriola-Villalobos P., Benitez-Del-Castillo J.M., Garcia-Feijoo J. (2017). Tear cytokine profile of glaucoma patients treated with preservative-free or preserved latanoprost. Ocul. Surf..

[112] Boimer C., Birt C.M. (2013). Preservative exposure and surgical outcomes in glaucoma patients: The PESO study. J. Glaucoma.

[113] Tomić M., Kaštelan S., Soldo K.M., Salopek-Rabatić J. (2013). Influence of BAK-preserved prostaglandin analog treatment on the ocular surface health in patients with newly diagnosed primary open-angle glaucoma. BioMed Res. Int..

[114] Huang C., Wang H., Pan J., Zhou D., Chen W., Li W., Chen Y., Liu Z. (2014). Benzalkonium chloride induces subconjunctival fibrosis through the COX-2-modulated activation of a TGF-β1/Smad3 signaling pathway. Investig. Ophthalmol. Vis. Sci..

[115] Broadway D.C., Grierson I., O’Brien C., Hitchings R.A. (1994). Adverse effects of topical antiglaucoma medication. II. The outcome of filtration surgery. Arch. Ophthalmol..

[116] Merani R., McPherson Z.E., Luckie A.P., Gilhotra J.S., Runciman J., Durkin S., Muecke J., Donaldson M., Aralar A., Rao A. (2016). Aqueous Chlorhexidine for Intravitreal Injection Antisepsis: A Case Series and Review of the Literature. Ophthalmology.

[117] Epstein N.E. (2021). Review: Perspective on ocular toxicity of presurgical skin preparations utilizing Chlorhexidine Gluconate/Hibiclens/Chloraprep. Surg. Neurol. Int..

[118] Green K., Livingston V., Bowman K., Hull D.S. (1980). Chlorhexidine Effects on Corneal Epithelium and Endothelium. Arch. Ophthalmol..

[119] Hamill M.B., Osato M.S., Wilhelmus K.R. (1984). Experimental evaluation of chlorhexidine gluconate for ocular antisepsis. Antimicrob. Agents Chemother..

[120] Dasgupta S. (2016). The course of dry eye following phacoemulsification and manual—SICS: A prospective study based on Indian scenario. Int. Eye Sci..

[121] Miyake K., Yokoi N. (2017). Influence on ocular surface after cataract surgery and effect of topical diquafosol on postoperative dry eye: A multicenter prospective randomized study. Clin. Ophthalmol..

[122] Kato K., Miyake K., Kondo N., Asano S., Takeda J., Takahashi A., Takashima Y., Kondo M. (2017). Conjunctival Goblet Cell Density Following Cataract Surgery with Diclofenac Versus Diclofenac and Rebamipide: A Randomized Trial. Am. J. Ophthalmol..

[123] Oh T., Jung Y., Chang D., Kim J., Kim H. (2012). Changes in the tear film and ocular surface after cataract surgery. Jpn. J. Ophthalmol..

[124] Yanai R., Yamada N., Ueda K., Tajiri M., Matsumoto T., Kido K., Nakamura S., Saito F., Nishida T. (2006). Evaluation of povidone-iodine as a disinfectant solution for contact lenses: Antimicrobial activity and cytotoxicity for corneal epithelial cells. Contact Lens Anterior Eye.

[125] Epstein S.P., Ahdoot M., Marcus E., Asbell P.A. (2009). Comparative toxicity of preservatives on immortalized corneal and conjunctival epithelial cells. J. Ocul. Pharmacol. Ther..

[126] Hwang H.B., Kim H.S. (2014). Phototoxic effects of an operating microscope on the ocular surface and tear film. Cornea.

[127] Ipek T., Hanga M.P., Hartwig A., Wolffsohn J., O’Donnell C. (2018). Dry eye following cataract surgery: The effect of light exposure using an in-vitro model. Contact Lens Anterior Eye.

[128] Ram J., Gupta A., Brar G., Kaushik S., Gupta A. (2002). Outcomes of phacoemulsification in patients with dry eye. J. Cataract. Refract. Surg..

[129] Kim J., Kim M.K., Ha Y., Paik H.J., Kim D.H. (2021). Improved accuracy of intraocular lens power calculation by preoperative management of dry eye disease. BMC Ophthalmol..

[130] Trattler W.B., Majmudar P.A., Donnenfeld E.D., McDonald M.B., Stonecipher K.G., Goldberg D.F. (2017). The Prospective Health Assessment of Cataract Patients’ Ocular Surface (PHACO) study: The effect of dry eye. Clin. Ophthalmol..

[131] Epitropoulos A.T., Matossian C., Berdy G.J., Malhotra R.P., Potvin R. (2015). Effect of tear osmolarity on repeatability of keratometry for cataract surgery planning. J. Cataract. Refract. Surg..

[132] Yang F., Yang L., Ning X., Liu J., Wang J. (2024). Effect of dry eye on the reliability of keratometry for cataract surgery planning. J. Fr. Ophtalmol..

[133] Hiraoka T., Asano H., Ogami T., Nakano S., Okamoto Y., Yamada Y., Oshika T. (2022). Influence of Dry Eye Disease on the Measurement Repeatability of Corneal Curvature Radius and Axial Length in Patients with Cataract. J. Clin. Med..

[134] Kojima T. (2018). Contact Lens-Associated Dry Eye Disease: Recent Advances Worldwide and in Japan. Investig. Ophthalmol. Vis. Sci..

[135] Arita R., Itoh K., Inoue K., Kuchiba A., Yamaguchi T., Amano S. (2009). Contact lens wear is associated with decrease of meibomian glands. Ophthalmology.

[136] Nagahara Y., Koh S., Oshita Y., Nagano T., Mano H., Nishida K., Watanabe H. (2017). Diquafosol Ophthalmic Solution Increases Pre- and Postlens Tear Film During Contact Lens Wear in Rabbit Eyes. Eye Contact Lens.

[137] Yang B., Ma X., Liu L., Cho P. (2021). Vision-related quality of life of Chinese children undergoing orthokeratology treatment compared to single vision spectacles. Contact Lens Anterior Eye.

[138] Willcox M.D.P., Argüeso P., Georgiev G.A., Holopainen J.M., Laurie G.W., Millar T.J., Papas E.B., Rolland J.P., Schmidt T.A., Stahl U. (2017). TFOS DEWS II Tear Film Report. Ocul. Surf..

[139] Miao C.X., Xu X.Y., Zhang H. (2017). Analysis of corneal complications in children wearing orthokeratology lenses at night. Zhonghua Yan Ke Za Zhi.

[140] De Paiva C.S., Chen Z., Koch D.D., Hamill M.B., Manuel F.K., Hassan S.S., Wilhelmus K.R., Pflugfelder S.C. (2006). The incidence and risk factors for developing dry eye after myopic LASIK. Am. J. Ophthalmol..

[141] Nair S., Kaur M., Sharma N., Titiyal J.S. (2023). Refractive surgery and dry eye—An update. Indian J. Ophthalmol..

[142] Liu Y.C., Yam G.H., Lin M.T., Teo E., Koh S.K., Deng L., Zhou L., Tong L., Mehta J.S. (2021). Comparison of tear proteomic and neuromediator profiles changes between small incision lenticule extraction (SMILE) and femtosecond laser-assisted in-situ keratomileusis (LASIK). J. Adv. Res..

[143] Konomi K., Chen L.L., Tarko R.S., Scally A., Schaumberg D.A., Azar D., Dartt D.A. (2008). Preoperative characteristics and a potential mechanism of chronic dry eye after LASIK. Investig. Ophthalmol. Vis. Sci..

[144] Cohen E., Spierer O. (2018). Dry Eye Post-Laser-Assisted In Situ Keratomileusis: Major Review and Latest Updates. J. Ophthalmol..

[145] Jung J.W., Kim J.Y., Chin H.S., Suh Y.J., Kim T.I., Seo K.Y. (2017). Assessment of meibomian glands and tear film in post-refractive surgery patients. Clin. Exp. Ophthalmol..

[146] Uchino M., Yokoi N., Shimazaki J., Hori Y., Tsubota K., on behalf of the Japan Dry Eye Society (2022). Adherence to Eye Drops Usage in Dry Eye Patients and Reasons for Non-Compliance: A Web-Based Survey. J. Clin. Med..

[147] Teo C.H.Y., Ong H.S., Liu Y.C., Tong L. (2020). Meibomian gland dysfunction is the primary determinant of dry eye symptoms: Analysis of 2346 patients. Ocul. Surf..

[148] Yang B., Wen K., Li J., Zhang S., Fan Z., Liang X., Liang L. (2021). Quantitative evaluation of lipid layer thickness and blinking in children with allergic conjunctivitis. Graefes Arch. Clin. Exp. Ophthalmol..

